# High Levels of BMP2 Promote Liver Cancer Growth via the Activation of Myeloid-Derived Suppressor Cells

**DOI:** 10.3389/fonc.2020.00194

**Published:** 2020-03-04

**Authors:** Gui Wu, Fei Huang, Yaoqing Chen, Yuehong Zhuang, Yunpeng Huang, Yun Xie

**Affiliations:** ^1^Department of Orthopedics, First Affiliated Hospital, Fujian Medical University, Fuzhou, China; ^2^Central Lab, First Affiliated Hospital of Fujian Medical University, Fuzhou, China; ^3^Department of Human Anatomy and Embryology, Institute of Neuroscientific Study, Fujian Medical University, Fuzhou, China

**Keywords:** BMP2, liver cancer, MDSCs, IL6, cell proliferation

## Abstract

Bone morphogenetic protein 2 (BMP2) signaling had significant roles in diverse pathological processes, such as cancer. Nevertheless, the interaction between BMP2 and carcinoma development remained largely unknown. In particular, the roles that BMP2 play in the development of liver cancer remained controversial, and mechanisms were unclear. BMP2 with strong osteogenic potential had been manufactured into various bone materials. However, cancer risk concerns were raised in recent years. Thus, we focused on analyzing the effects of exogenous BMP2 on the growth of liver cancer and the detailed mechanisms. We found that both intravenous injection of rhBMP2 and *in vivo* implantation of rhBMP2 materials could lead to the expansion of myeloid-derived suppressor cells (MDSCs) in peripheral blood and subsequently enhanced the infiltration of MDSCs into tumor *in vivo*. Furthermore, BMP2 signaling-activated MDSCs could secrete IL6 to enhance cell proliferation of liver cancer cells *in vitro* and facilitate liver cancer growth *in vivo*. Our study indicated that increased concentration of BMP2 within the peripheral blood could enhance liver cancer growth via the activation of MDSCs. In this study, the roles that BMP2 played in liver cancer growth were further confirmed and the detailed mechanisms about how BMP2 enhanced liver cancer growth were also elucidated.

## Introduction

Bone morphogenetic protein (BMP), which belonged to the transforming growth factor β family, was discovered by Urist in 1965 (1). Recombined human bone morphogenetic protein 2 (rhBMP2), as a type of BMP molecule, displayed strong osteogenic potential, which could induce bone formation even in ectopic sites. Although many studies had reported the satisfactory outcomes of rhBMP2 in the induction of new bone regeneration, several complications of rhBMP2, such as postoperative radiculitis, ectopic bone formation, edema, and especially carcinogenesis (2), raised some concerns on safety issues. Remarkably, in 2010, the US Food and Drug Administration reported a notably increased cancer rate (4-fold) among patients in the rhBMP2-treated group compared with the control group at the second year (2). However, the interaction between rhBMP2 and heterotopic tumors remained largely unknown, which might potentially threaten the patients' health. Thus, we focused on the roles of exogenous BMP2 playing in carcinoma progression.

BMP2 had been reported to be involved in the tumorigenesis and the progression of cancer (3). However, functions of BMP2 signaling in carcinoma development were controversial. Activation of BMP2/4 signaling could inhibit the self-renewal but enhanced the differentiation of cancer stem cells in colon cancer and further promoted the sensitivity of colon cancer cells to chemotherapy (2, 4, 5). However, BMPs could also induce the epithelial-to-mesenchymal transition (EMT) of colon cancer cells, resulting in the promoted growth and metastasis of cancer (6, 7). Moreover, BMP signaling was also highly expressed in breast cancer, prostate cancer, and pancreatic cancer, which might be closely related to the malignant progression of those tumors (8, 9). TGF-β was a classical EMT stimulator. BMPs also played a role in the induction of EMT in many tumors as they were members of the TGF-β superfamily (10, 11). More importantly, BMP6 signaling could activate myeloid-derived suppressor cells (MDSCs) to secrete IL10 and could promote the differentiation of M2 macrophages, which could enhance the invasion and metastasis of tumor cells (12).

Liver cancer with characteristics of a high degree of malignancy, rapid progression, poor prognosis, and a high mortality rate seriously threatened human health and life (13). However, the function of BMP signaling in the progression of liver cancer was rarely studied (14). It was reported that BMP Receptor I (BMPRIA) was highly expressed in liver cancer cells, and knockdown of BMP2 in liver cancer cells could suppress the migration and invasion of liver cancer (15). However, other studies have also shown that BMP2 signaling could inhibit the proliferation and migration of liver cancer cells (16). Therefore, considering the potential tumorigenesis concerns of rhBMP2 used in clinics and the uncertainty of roles that BMP2 played in the progression of liver cancer, we mainly focused on the roles that BMP2 play in liver cancer growth.

We analyzed the public clinical database of Roessler Liver, KM plotter, and TCGA, and found that high BMP2 expression indicated poor prognosis of human liver cancer. Moreover, in our results, high levels of rhBMP2 concentration within the peripheral blood could enhance the growth of subcutaneous liver tumors in mouse models. Besides, local delivery of rhBMP2 collagen gels to the muscle punch of mice, which imitated the clinical application of rhBMP2 materials, could increase rhBMP2 concentration within the peripheral blood, and finally enhanced liver cancer growth *in vivo*. In our mouse models, high concentration of BMP2 within the peripheral blood could promote the differentiation of MDSCs from bone marrows and the infiltration of MDSCs to tumor tissues. MDSCs could subsequently secrete IL6 to enhance the proliferation of liver cancer cells both *in vitro* and *in vivo*. In this study, the roles that BMP2 played in liver cancer growth were further confirmed and the detailed mechanisms about how BMP2 enhanced the liver cancer growth were also elucidated.

## Materials and Methods

### Antibodies and Reagents

The following antibodies were used in this study: anti-CD11b-FITC (Biolegend, 101206, dilution ratio: 1:100 for flow cytometry and immunofluorescence); anti-Ly-6G-PE (eBioscience, 12-9668-82, dilution ratio: 1:100 for flow cytometry); anti-Ly-6C-PE (Cell eBioscience, 12-5932-82, dilution ratio: 1:100 for flow cytometry); anti-p-Smad1/5 (Cell Signaling Technology, 9516, dilution ratio: 1:100 for immunofluorescence and 1:1000 for Western blot); anti-Smad1/5 (Cell Signaling Technology, 6,944, dilution ratio: 1:100 for immunofluorescence and 1:1000 for Western blot); anti-p-Stat3 (Cell Signaling Technology, 9,145, dilution ratio: 1:1000 for Western blot); anti-Stat3 (Cell Signaling Technology, 9,139, dilution ratio: 1:1000 for Western blot); anti-p-Erk (Cell Signaling Technology, 4,370, dilution ratio: 1:1000 for Western blot); anti-Erk (Cell Signaling Technology, 4,695, dilution ratio: 1:1000 for Western blot); anti-mouse IL6 (Biolegend, 504501, dilution ratio: 1:500 for neutralization); anti-S100A9 (Abcam, GB111149, dilution ratio: 1:2000 for IHC); anti-Ki67 (Servicebio, GB13030-2, dilution ratio: 1:500 for IHC), and anti-β-actin (Sigma, A1978, dilution ratio: 1:1000 for Western blot). Rat tail collagen I (Corning, 356236) and RhBMP2 (R&D, 355-BM-100) were also used. Tris–HCl, NaCl, and other chemicals were from Sigma.

### RhBMP2 Collagen Gel Preparation and Characterization

RhBMP2 was dissolved in PBS to obtain a concentration of 1 mg/ml. For each rhBMP2 collagen gel, 7.5 μl of rhBMP2 solution was added to 150 μl of rat tail collagen I solution (3%, w/v) and mixed evenly, while in the control group, only 7.5 μl of PBS was added. Thereafter, another 3 μl of 1 M NaOH was added to the above solution, and pH value was adjusted to 7.0. Collagen solution with or without rhBMP2 was stored at 37°C for 2 h to form collagen gels. Then, collagen gels were freeze dried and kept at room temperature until use. The microstructures of rhBMP2 collagen gels were observed by a scanning electron microscopy (SEM, S-2400; Hitachi, Japan).

### Mice

ICR and C57BL/6 mice used in this study were bred and maintained in a specific pathogen-free animal facility at Fujian Medical University. Mice were euthanized by cervical dislocation after anesthesia. All animal experiments were approved by the Animal Ethical Committee of Fujian Medical University (2018-039).

### Tumorigenesis

All experiments were performed with mycoplasma-free cells. 5 × 10^5^ H22 or Hepa1-6 cells were injected subcutaneously into the abdomen of each ICR or C57BL/6 mice to make the subcutaneous tumorigenesis mice model. Tumor sizes were measured by tumor length and width using a clipper and then tumor volume was calculated using the formula *V* = (*L* × *W* × *W*)/2, where *V* is tumor volume, *W* is tumor width, and *L* is tumor length. For the injection model, 100 μg/kg rhBMP2 was injected into the tail veins of mice three times per week. For the local implantation model, 15 μg of rhBMP2 was imbedded into 300 μg collagen for transplantation per mouse.

### Mouse Peripheral Blood Samples

Heparin anti-coagulated peripheral blood (PB) from mouse tail veins was diluted 1:2 (vol:vol) in RPMI-1640 with 5% FBS. The diluted whole blood samples were collected. After RBCs (red blood cells) were lysed by lysing solution for 5 min at room temperature, the white blood cells (WBCs) were washed with PBS. Then, WBCs were subjected to immunostaining for flow cytometry.

### Immunostaining for Flow Cytometry

Mouse PB samples or bone marrow samples were resuspended in PBS with 2% FBS. The surface molecules CD11b, Ly-6G, and Ly-6C were stained with antibodies for 30 min at 4°C. Flow cytometric analysis was performed using BD FACS C6 Flow Cytometer. The results were analyzed by the software FlowJo 7.6.1.

### Immunofluorescence for Tissues

The tissues were embedded in paraffin to be cut into 3 μm tissue sections. Tissue sections were dewaxed with xylene. Then, sections were rehydrated with 100–95–75% alcohol gradients. After antigens were repaired in 0.01 M citrate buffer (pH = 6.0) at 95°C for 15 min, the sections were stained with the CD11b-FITC antibody and p-Smad1/5 antibody overnight at 4°C. On the second day, cells and sections were incubated with TRITC-conjugated secondary antibody for 1 h at room temperature. DAPI (Solarbio, C0060) was used to stain the nucleus. Then, sections were mounted with anti-quencher (Beyotime, P0128). Images were taken by Olympus microscope BX53.

### Culture and Activation of MDSCs *in vitro*

Mouse bone marrow cells were harvested to be a single-cell suspension in RPMI-1640 with 5% FBS. After RBCs were lysed, the bone marrow cells were resuspended in complete RPMI-1640 with 2% FBS at 10^6^/ml. 10 ng/ml GMCSF (R&D, 415-ML-005) and 20 ng/ml rhBMP2 (R&D, 355-BM-100) were added as grouped.

### CCK-8 Cell Proliferation Assay

About 3000 cells were cultured in each well of 96-well plates with 20 ng/ml rhBMP2, 2 ng/ml IL6, or 1 μg/ml neutralized IL6 antibody with 2% FBS containing medium. Relative cell intensity was measured with a cell-counting kit (CCK-8, Dojindo Molecular Technologies) after indicated times.

### Matrigel 3D Cultures

Cells in 2D cultures were trypsinzed and resuspended in media, and 20,000 cells were plated on each 100 μl of Matrigel (BD BioSciences, New Jersey, USA). Media containing 10% FBS was added for cell growth.

### ELISA Assay

Tumor tissues were homogenized in PBS to be samples. All samples originated from tumors were quantified to the concentration 10 mg/ml by BCA assay. The BCA kit was from Beyotime (P0009). Samples from tumors or mouse peripheral blood were subjected to BMP2 ELISA assay (Sigma-Aldrich Co., USA, C9879) or IL6 ELISA assay (MultiSciences Biotech, CO., LTD 70-EK206HS-96).

### Quantitative Real-Time PCR

Total cell RNA was isolated with TRIzol (Invitrogen), and cDNA was synthesized with Revertra Ace (Promega, Madison, USA). Real-time PCR was performed with an ABI QuantStudio 5 system. The expression level of genes was measured using the comparative Ct method. Expression values were normalized to β-actin expression. The primer sequences were: Arg1: Forward: 5′-CTCCAAGCCAAAGTCCTTAGAG-3′; Reverse: 5′-AGGAGCTGTCATTAGGGACATC-3′. iNOS: Forward: 5′-GGAGCATCACCCCTGTGT-3′; Reverse: 5′-GGTCTTCCAGGGCTCGAT-3′. IL6: Forward: 5′-TCTATACCACTTCACAAGTCGGA-3′; Reverse: 5′-GAATTGCCATTGCACAACTCTTT-3′. IL8: Forward: 5′-TCGAGACCATTTACTGCAACAG-3′; Reverse: 5′-CATTGCCGGTGGAAATTCCTT-3′. IL10: Forward: 5′-GCTCTTACTGACTGGCATGAG-3′; Reverse: 5′-CGCAGCTCTAGGAGCATGTG-3′. β-Actin: Forward: 5′-ACCAACTGGGACGATATGGAGAAGA-3′; Reverse: 5′-TACGACCAGAGGCATACAGGGACAA-3′.

### Immunoblotting

Cells were lysed with TNE buffer (10 mM Tris–HCl, 150 mM NaCl, 1 mM EDTA, 0.5% NP40, pH = 7.5). For immunoblotting assay, cell lysates were mixed with 4 × loading buffer (40 mM Tris–HCl, 200 mM DTT, 4% SDS, 40% Glycerol, and 0.032% Bromophenol Blue, pH = 8.0). The samples were run with 4% stacking gel and 10% separating gels. Then, proteins on the gels were transferred to nitrocellulose filter membranes for antibodies incubated. The membranes' exposure was done with Thermo Pierce ECL and FluorChem E (ProteinSimple).

### Statistical Analysis

Student's *t*-test, two-way ANOVA, one-way ANOVA test, Wilcox rank sum test, and log-rank test were used. *P* < 0.05 was considered statistically significant.

## Results

### High Expression of BMP2 Indicated Poor Prognosis in Human HCC

**Figure 1:**
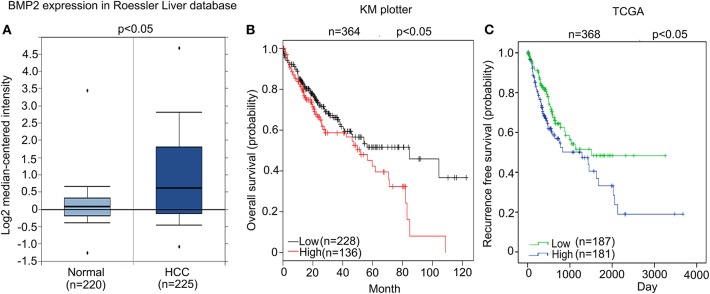
High expression of BMP2 indicated poor prognosis in human HCC. **(A)** BMP2 expression was higher in human HCC than in normal liver tissues. The results shown here were analyzed by Oncomine Research Network. **(B)**. Higher BMP2 expression indicates worse prognosis in human HCC. Kaplan–Meier plots of HCC patients, stratified by expression of BMP2. Data obtained from the Kaplan–Meier plotter database. The *p*-value was calculated by a log-rank test. **(C)** Higher BMP2 expression indicates worse prognosis in human HCC. Kaplan–Meier plots of HCC patients, stratified by expression of BMP2. Data obtained from the TCGA Research Network. The *p*-value was calculated by a log-rank test.

We firstly analyzed whether high expression of BMP2 influenced the prognosis of human HCC. From the public clinical microarray database of Roessler Liver, we found that the expression of BMP2 in HCC tissues was higher than that of normal liver tissues (Figure 1A) (17). Moreover, survival analysis based on the public clinical database of KM plotter from 364 liver cancer patients was carried out. Patients were divided into a high BMP2 expression group (*n* = 136) and a low BMP2 expression group (*n* = 228) by the median expression of BMP2 mRNA. The overall survival of HCC patients with high BMP2 expression was poorer than that of patients with low BMP2 expression (Figure 1B) (18). The recurrence-free survival of HCC patients was also carried out based on the public clinical database of TCGA from 368 patients (Figure 1C). Patients were divided into a high BMP2 expression group (*n* = 181) and a low BMP2 expression group (*n* = 187) by the mean expression of BMP2 mRNA. Same with the overall survival, the recurrence-free survival of HCC patients with high BMP2 expression was also poor (Figure 1C). The results above indicated that high expression of BMP2 resulted in poor prognosis in human HCC.

### Increased Concentration of rhBMP2 Within Peripheral Blood Promoted the Growth of Liver Cancer *in vivo*

**Figure 2:**
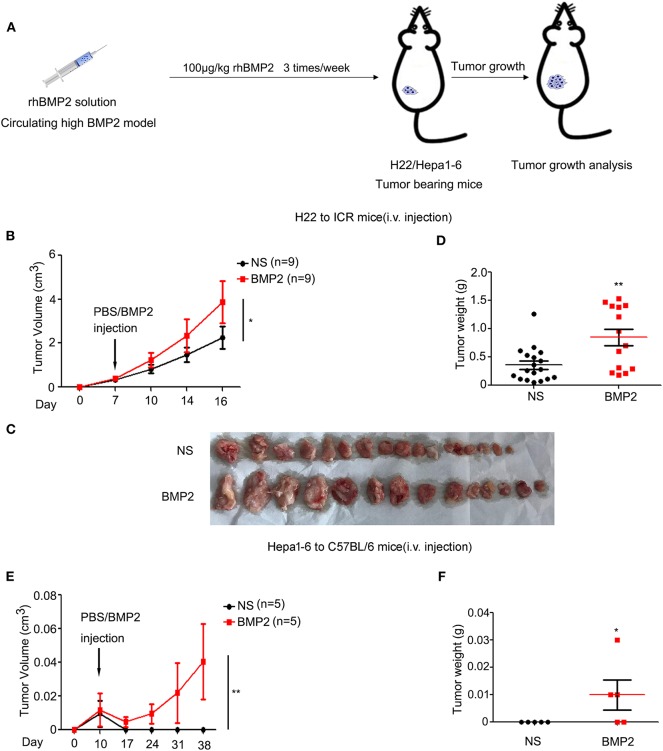
Increased concentration of rhBMP2 within peripheral blood promoted the growth of liver cancer *in vivo*. **(A)** Flow diagram of experiments. BMP2 solution was intravenously injected into H22/Hepa1-2 tumor-burdened mice to study the effects of circulating high BMP2 concentration on the growth of liver cancers. The tumor volumes were measured. **(B)** The average tumor volumes of subcutaneous H22 tumors in ICR mice after intravenous injection. Data were expressed as means ± SEM (*n* = 9). “*n*” indicated the number of mice used. *P < 0.05. **(C)** ICR mice in **(A)** were sacrificed and tumor tissues were harvested. The digital image of tumor tissues in NS and rhBMP2 groups was displayed. **(D)** ICR mice in **(A)** were sacrificed and tumor tissues were harvested. Tumor weights of NS and rhBMP2 group were compared. *P < 0.05, **P < 0.01. **(E)** The average tumor volumes of subcutaneous Hepa1-6 tumors in C57BL/6 mice after intravenous injection. Data were expressed as means ± SEM (*n* = 5). “*n*” indicated the number of mice used. **P < 0.01. **(F)** C57BL/6 ICR mice in **(A)** were sacrificed and tumor tissues were harvested. Tumor weights of NS and rhBMP2 group were compared. *P < 0.05.

As high BMP2 expression indicated poor prognosis in human liver cancer, we went further to study whether the intravenous delivery of rhBMP2 could promote the growth of liver cancer in mouse models. H22 or Hepa1-6 liver cancer cells were subcutaneously injected into ICR or C57BL/6 mice. On the 7th day, mice were separated into two groups randomly. Then, rhBMP2 solution was directly injected through the tail veins of ICR and C57BL/6 mice three times per week (Figure 2A). Both H22 and Hepa1-6 tumor volumes and tumor weights were compared between the control and the BMP2 group. The volumes of subcutaneous H22 tumors in ICR mice were measured every 3–4 days. We found that intravenous injection of rhBMP2 could accelerate the growth of H22 tumors in ICR mice (Figures 2B,C). Consistently, the weights of H22 tumors were also significantly larger in rhBMP2-treated mice than in control mice at the end of observation (Figure 2D). Differently, the growth of Hepa1-6 tumors in C57BL/6 mice was slower than H22 tumors in ICR mice in general; thus, the tumor volumes were recorded every week. We found that direct intravenous injection of rhBMP2 could facilitate the growth of Hepa1-6 tumor in C57BL/6 mice (Figures 2E,F). Therefore, increased concentration of rhBMP2 in the circulation system could promote the growth of liver cancers.

### RhBMP2 Collagen Gels Promoted the Growth of Liver Cancer *in vivo*

**Figure 3:**
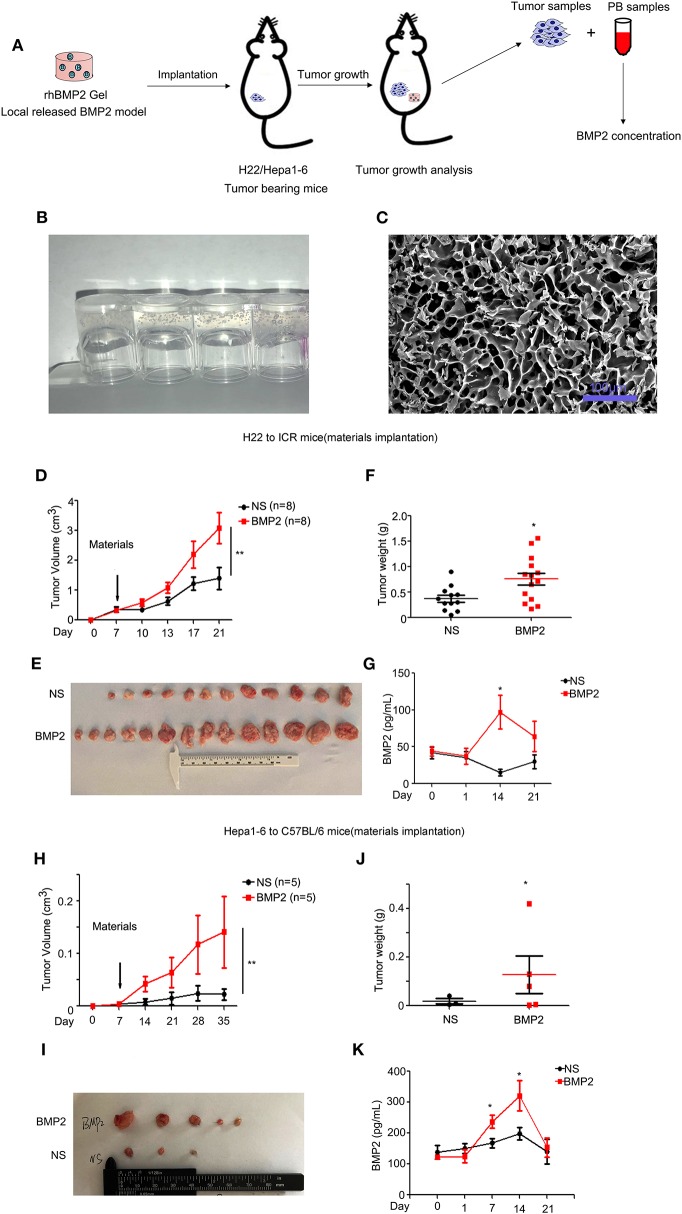
RhBMP2 collagen gels promoted the growth of liver cancer *in vivo*. **(A)** Flow diagram of experiments. The rhBMP2 collagen gels were implanted within the muscle pouch of H22/Hepa1-2 tumor-burdened mice to observe the effects of local delivered rhBMP2 on the growth of liver cancers. The tumor volumes were measured. **(B)** The digital photograph of rhBMP2 collagen gels. The containers were placed upside down. **(C)** The electron microscopy image of rhBMP2 collagen gels after the gels were freeze dried. Scale bar: 100 μm. **(D)** The average tumor volumes of subcutaneous H22 tumors in ICR mice after the material implantation. Data were expressed as means ± SEM (*n* = 8). “*n*” indicated the number of mice used. **P < 0.01. **(E)** ICR mice in **(A)** were sacrificed and tumor tissues were harvested. The digital image of subcutaneous H22 tumors was displayed. **(F)** ICR mice in **(A)** were sacrificed and tumor tissues were harvested. Tumor weights of NS and rhBMP2 group were compared. *P < 0.05. **(G)** RhBMP2 concentration in peripheral blood of ICR mice at each time point for NS and rhBMP2 groups, and the results of each time point were compared. *P < 0.05. **(H)** The average tumor volumes of subcutaneous Hepa1-6 tumors in C57BL/6 mice after the material implantation. Data were expressed as means ± SEM (*n* = 5). “*n*” indicated the number of mice used. **P < 0.01. **(I)** C57BL/6 mice in **(A)** were sacrificed and tumor tissues were harvested. The digital image of subcutaneous Hepa1-6 tumors was displayed. **(J)** C57BL/6 mice in **(A)** were sacrificed and tumor tissues were harvested. Tumor weights of NS and rhBMP2 group were compared. *P < 0.05. **(K)** RhBMP2 concentration in peripheral blood of C57BL/6 mice at each time point for NS and rhBMP2 groups, and the results of each time point were compared. *P < 0.05.

Due to the safety concerns of rhBMP2/ACS materials used in tumor patients, we went further to analyze whether rhBMP2 materials enhanced the growth of liver cancer *in vivo* (Figure 3A). In this study, collagen gels were used as carriers for rhBMP2 to mimic the functions of rhBMP2 materials. The rat tail collagen I solution was successfully coagulated after alkaline liquid treatment (Figure 3B), and rhBMP2 was embedded in the collagen. After it was freeze dried, a collagen sponge with or without rhBMP2 was obtained. Many porous structures with a pore size around 10–40 μm within the collagen could be found under scanning electron microscopy (Figure 3C).

To investigate the effects of rhBMP2 collagen gels on the growth of liver cancer, mice were inoculated with H22 and Hepa1-6 cancer cells, respectively. On the 7th day, mice were separated into two groups randomly. Then, rhBMP2 collagen gels were implanted in the hip muscle pouch of mice, while only empty collagen gels were implanted in the control group (Figure 3A). The volumes of subcutaneous H22 tumors in ICR mice were measured every 3–4 days, and we found that rhBMP2 collagen gels could promote the growth of H22 tumors in ICR mice (Figures 3D,E). At day 21, mice treated with rhBMP2 collagen gels resulted in significantly larger tumors compared with the control group (*P* < 0.05), and the result was in agreement with that of tumor weights (Figure 3F). The peripheral blood samples were collected to measure the plasma BMP2 concentration of ICR mice. As expected, local implantation of rhBMP2 collagen gels could lead to the blood release of rhBMP2 and resulted in increased plasma rhBMP2 concentration in ICR mice. The peak concentration values were observed at day 14 (Figure 3G). The growth of Hepa1-6 tumors in C57BL/6 mice was slower than H22 tumors in ICR mice in general; thus, the tumor volumes were recorded every week. We showed that rhBMP2 treatment could accelerate the growth of Hepa1-6 tumors in mice and the results were more significant at day 28 and day 35 (Figures 3H–J). The mean BMP2 concentration for C57BL/6 mice was elevated after the implantation of rhBMP2 materials. The peak concentration values were also observed at day 14 (Figure 3K). Results above indicated that local delivery of rhBMP2 collagen gels to the muscle punch of mice could increase the rhBMP2 concentration within the peripheral blood to enhance the liver cancer growth.

### BMP2 Promoted the Expansion of MDSCs in Peripheral Blood and the Infiltration of MDSCs Into Tumor *in vivo*

**Figure 4:**
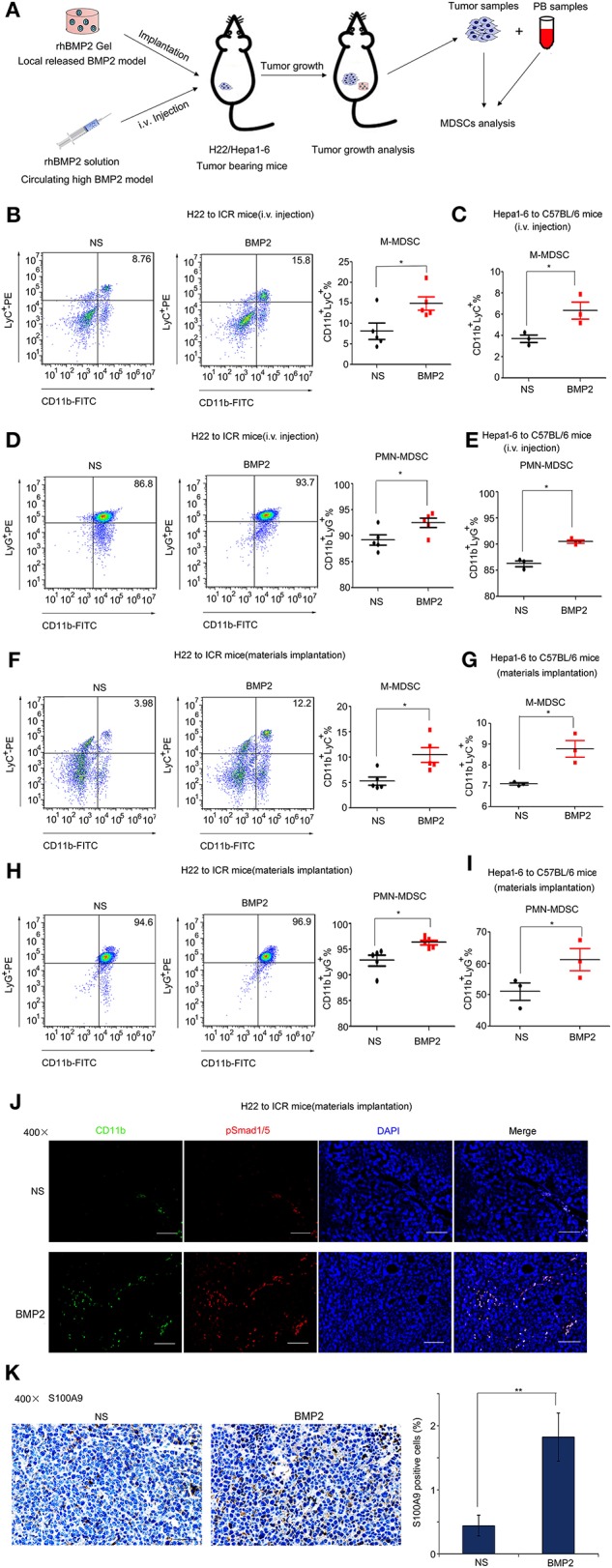
BMP2 promoted the expansion of MDSCs in peripheral blood and the infiltration of MDSCs into tumors. **(A)** Flow diagram of experiments. The BMP2 solution was intravenously injected to H22/Hepa1-2 tumor-burdened mice and rhBMP2 collagen gels were implanted within the muscle pouch of H22/Hepa1-2 tumor-burdened mice. The tumor tissues were harvested and blood samples were collected for analyses. **(B)** Representative flow cytometric images and quantitative analysis of monocyte-derived MDSCs from rhBMP2 solution intravenously injected ICR mice. Cells from peripheral blood samples of ICR mice were stained with indicated antibodies and subjected to flow cytometric analyses. *P < 0.05. **(C)** Quantitative analyses of monocyte-derived MDSCs from rhBMP2 solution intravenously injected C57BL/6 mice. Cells from peripheral blood samples of C57BL/6 mice were stained with indicated antibodies and subjected to flow cytometric analyses. *P < 0.05. **(D)** Representative flow cytometric images and quantitative analysis of polymorphonuclear-derived MDSCs from rhBMP2 solution intravenously injected ICR mice. Cells from peripheral blood samples of ICR mice were stained with indicated antibodies and subjected to flow cytometric analyses. *P < 0.05. **(E)** Quantitative analyses of polymorphonuclear-derived MDSCs from rhBMP2 solution intravenously injected C57BL/6 mice. Cells from peripheral blood samples of C57BL/6 mice were stained with indicated antibodies and subjected to flow cytometric analyses. *P < 0.05. **(F)** Representative flow cytometric images and quantitative analysis of monocyte-derived MDSCs from rhBMP2 collagen gel-implanted ICR mice. Cells from peripheral blood samples of ICR mice were stained with indicated antibodies and subjected to flow cytometric analyses. *P < 0.05. **(G)** Quantitative analyses of monocyte-derived MDSCs from rhBMP2 collagen gel-implanted C57BL/6 mice. Cells from peripheral blood samples of C57BL/6 mice were stained with indicated antibodies and subjected to flow cytometric analyses. *P < 0.05. **(H)** Representative flow cytometric images and quantitative analysis of polymorphonuclear-derived MDSCs from rhBMP2 collagen gel-implanted ICR mice. Cells from peripheral blood samples of ICR mice were stained with indicated antibodies and subjected to flow cytometric analyses. *P < 0.05. **(I)** Quantitative analyses of polymorphonuclear-derived MDSCs from rhBMP2 collagen gel-implanted C57BL/6 mice. Cells from peripheral blood samples of C57BL/6 mice were stained with indicated antibodies and subjected to flow cytometric analyses. *P < 0.05. **(J)** Immunofluorescence images of subcutaneous H22 tumor samples from rhBMP2 collagen gel-implanted ICR mice. Blue: DAPI, Green: CD11b-FITC, Red: Smad1/5/8-TRITC. Scale bar: 100 μm. **(K)** Immunohistochemistry images indicated the expression of S100A9 of subcutaneous H22 tumor samples from rhBMP2 collagen gel-implanted ICR mice. Scale bar: 50 μm. Average percentages of S100A9-positive cells of at least three fields were shown on the right. **P < 0.01.

Monocytic and granulocytic myeloid-derived suppressor cells (M-MDSCs and PMN-MDSCs, respectively) that were derived from bone marrows were major myeloid populations associated with tumor development (19, 20). BMP2 had been reported to enhance the M1 to M2 macrophage transformation (21–23). Although BMP6 had been reported to activate MDSCs to secrete IL10 (12), the regulatory roles that BMP2 play in MDSC expansion remained unclear. Therefore, we went further to study the impact of rhBMP2 on the expansion of MDSCs and the infiltration of MDSCs into tumor (Figure 4A). Flow cytometry was used for quantitative analysis of M-MDSCs and PMN-MDSCs in PB. We found that intravenous delivery of rhBMP2 could promote the expansion of M-MDSCs and PMN-MDSCs in ICR and C57BL/6 mice (Figures 4B–E and Supplementary Figure 1A). Moreover, local delivery of rhBMP2 collagen gels could also promote the expansion of M-MDSCs and PMN-MDSCs in ICR and C57BL/6 mice (Figures 4F–I and Supplementary Figure 1B). Furthermore, the infiltration of MDSCs and the activation of BMP2 signaling in tumor tissues were detected by immunofluorescence staining. CD11b or S100A9 labeled MDSCs, and p-smad1/5 labeled the activation of BMP2 signaling (19, 24–26). We demonstrated that implantation of rhBMP2 collagen gels promoted the activation of BMP2 signaling in MDSCs and the infiltration of MDSCs into liver cancer tissues of ICR mice (Figures 4J,K and Supplementary Figures 2A,B).

### BMP2 Promoted the Expansion of MDSCs *in vitro*

**Figure 5:**
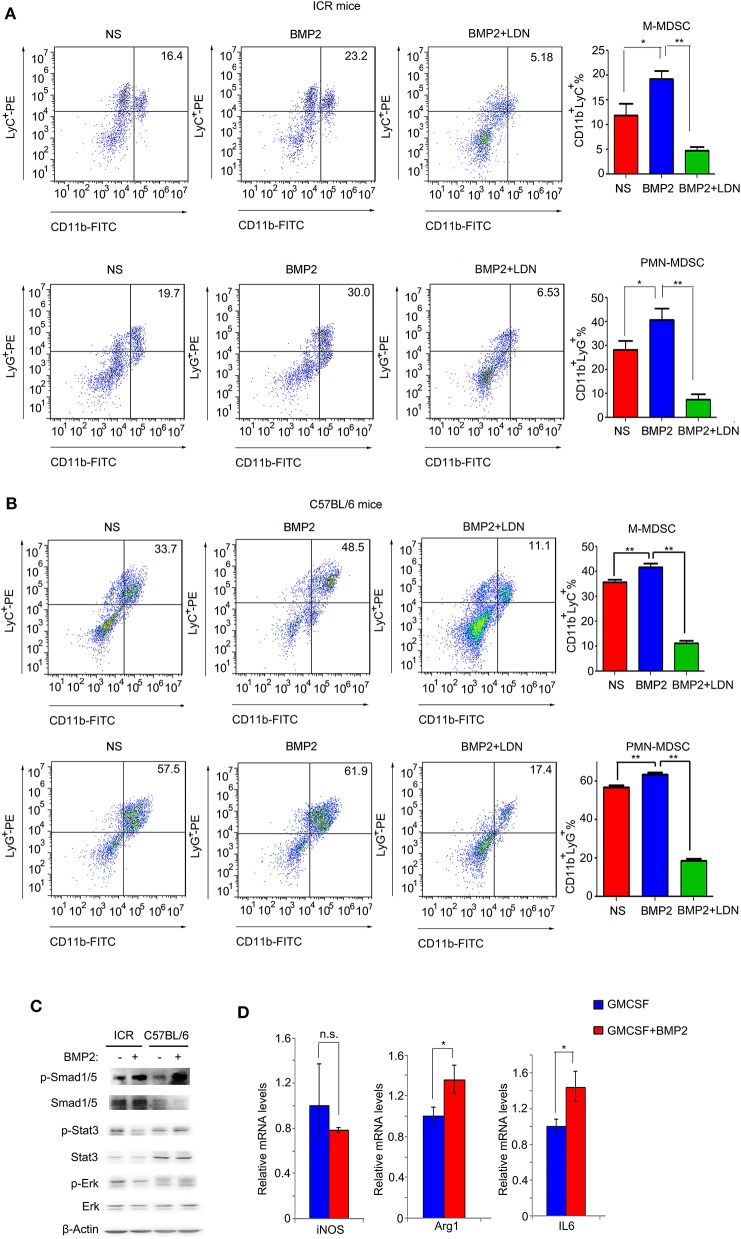
BMP2 promoted the expansion of MDSCs *in vitro*. **(A)** Representative flow cytometric images and quantitative analyses of monocyte-derived MDSCs and polymorphonuclear-derived MDSCs. Cells from bone marrow of ICR mice were stimulated with rhBMP2 or rhBMP2 with LDN-193189 *in vitro* and then stained with indicated antibodies and subjected to flow cytometric analyses. *P < 0.05, **P < 0.01. **(B)** Representative flow cytometric images and quantitative analyses of monocyte-derived MDSCs and polymorphonuclear-derived MDSCs. Cells from bone marrow of C57BL/6 mice were stimulated with rhBMP2 or rhBMP2 with LDN-193189 *in vitro*, and then stained with indicated antibodies and subjected to flow cytometric analyses. *P < 0.05, **P < 0.01. **(C)** Phosphorylation levels of Smad1/5, Stat3, and Erk after the bone marrow of ICR and C57BL/6 mouse were treated with BMP2. Cell lysates were harvested to be subjected to Western blot. β-Actin was the reference for all the blots. Total Smad1/5, Stat3, and Erk were the references for the phosphorylated proteins. **(D)** Comparison of relative iNOS, Arg1, and IL6 mRNA levels of bone marrow cells treated with BMP2. *P < 0.05.

Results above indicated that up-regulation of BMP2 levels in peripheral blood resulted in the increase of MDSCs in both peripheral blood and tumors. Thus, BMP2 properly enhanced the expansion of MDSCs to promote tumor growth. We extracted bone marrow cells from ICR mice and C57BL/6 mice, and stimulated them with rhBMP2 *in vitro*. We found that both the populations of CD11b^+^LyC^+^ M-MDSCs and CD11b^+^LyG^+^ PMN-MDSCs were expanded when bone marrow cells underwent BMP2 treatment, indicating that BMP2 could promote the expansion of MDSCs (Figures 5A,B). Moreover, when BMP2 signaling was blocked by the BMPRI inhibitor LDN-193189 in bone marrow cells, the expansion of MDSCs was attenuated (Figures 5A,B). BMP2 signaling could regulate downstream target genes via canonical Smad1/5/8 signaling pathway or non-Smads signaling (27, 28). Stat3 signaling or Erk signaling had been reported to be activated when MDSCs were activated (29, 30). For bone marrow cells, we found that phosphorylated Smad1/5 could be induced by BMP2 signaling, but Stat3 signaling or Erk signaling could not be activated by BMP2 signaling (Figure 5C). We could also find phosphorylated Smad1/5 in the CD11b^+^ cells infiltrating into tumors *in vivo* (Figure 4J and Supplementary Figure 2A). Therefore, BMP2 signaling might activate MDSCs via canonical Smad1/5/8 signaling but not non-Smads signaling. Arg1 and iNOS were the markers of MDSC population activation, and the activated MDSCs could secrete IL6, IL8, and IL10 to enhance cancer progression (19, 29). In our results, we found that BMP2 signaling could enhance the expression of the Arg1 and IL6 in bone marrows, rather than iNOS, IL8, and IL10 (Figure 5D and Supplementary Figure 3), indicating that BMP2 inducing MDSCs expansion might enhance liver cancer growth via IL6.

### MDSC Expansion Induced by BMP2 Enhanced Liver Cancer Growth via IL6

**Figure 6:**
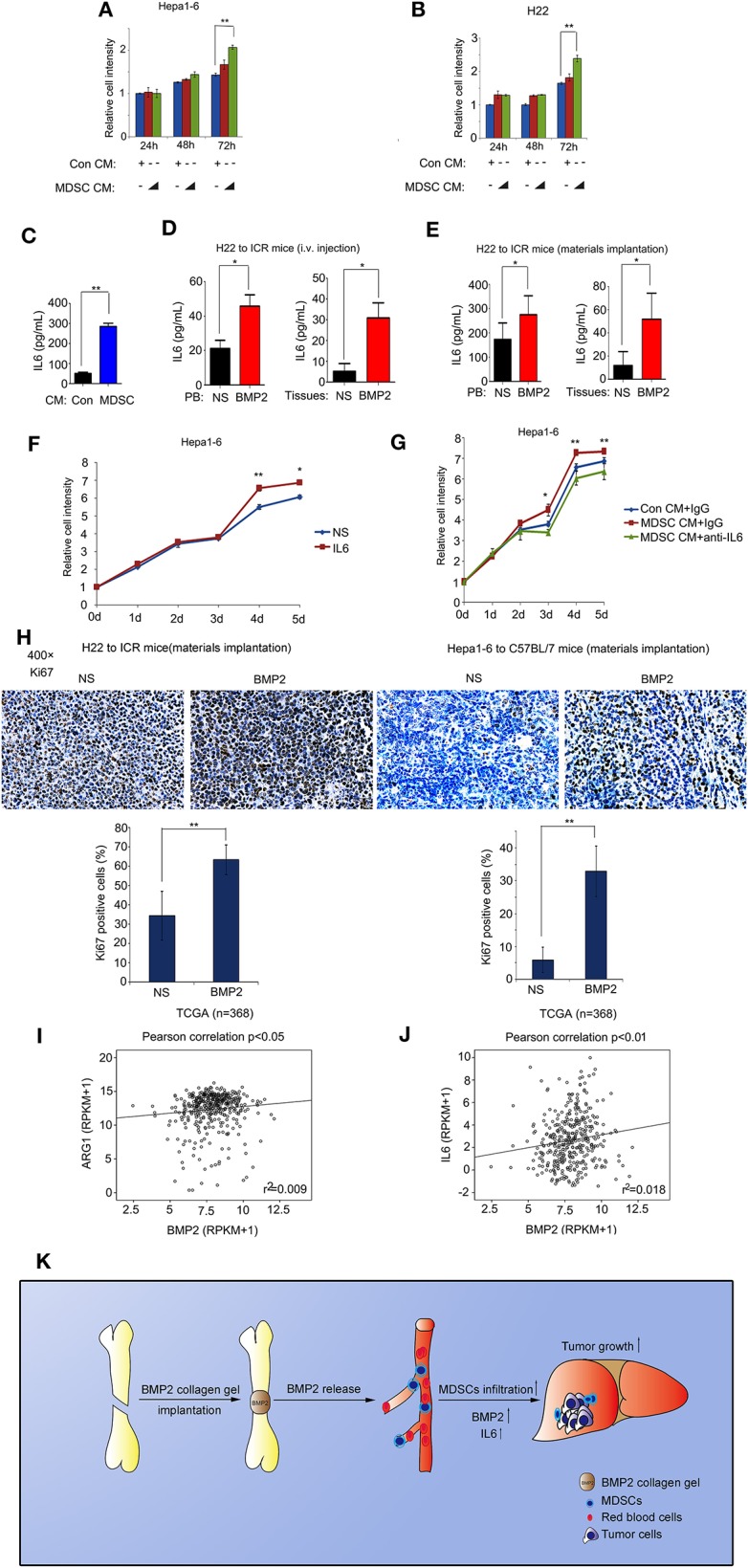
BMP2 activated MDSCs to enhance liver cancer growth via IL6. **(A)** About 3000 Hepa1-6 cells were cultured in each well of 96-well plates with control conditional medium or MDSC secreting conditional medium. Relative cell intensity was measured with a cell-counting kit after indicated time. **P < 0.01. **(B)** About 3000 H22 cells were cultured in each well of 96 well plates with control conditional medium or MDSC secreting conditional medium. Relative cell intensity was measured with a cell-counting kit after indicated time. **P < 0.01. **(C)** IL6 concentration from control conditional medium or MDSC secreting conditional medium were compared. **P < 0.01. **(D)** IL6 concentration in peripheral blood and tumor tissues of rhBMP2 solution intravenously injected ICR mice for NS and rhBMP2 groups were compared. *P < 0.05, **P < 0.01. **(E)** IL6 concentration in peripheral blood and tumor tissues of rhBMP2 collagen gels implanted ICR mice for NS and rhBMP2 groups were compared. *P < 0.05. **(F)** About 3000 Hepa1-6 cells were cultured in each well of 96-well plates with vehicle or IL6. Relative cell intensity was measured with a cell-counting kit after indicated time. *P < 0.05, **P < 0.01. **(G)** About 3000 Hepa1-6 cells were cultured in each well of 96-well plates with control conditional medium or MDSC secreting conditional medium. Control IgG or the anti-IL6 antibody were treated. Relative cell intensity was measured with a cell-counting kit after indicated time. *P < 0.05, **P < 0.01. **(H)** Immunohistochemistry images of ki67 subcutaneous H22 or Hepa1-6 tumor samples from rhBMP2 collagen gel-implanted ICR mice. Scale bar: 50 μm. Average percentages of Ki67-positive cells of at least three fields were shown below. **P < 0.01. **(I)** Pearson correlation of BMP2 and ARG1 expression were analyzed in human HCC. Data obtained from the TCGA Research Network. **(J)** Pearson correlation of BMP2 and IL6 expression were analyzed in human HCC. Data obtained from the TCGA Research Network. **(K)** Schematic diagram revealed the effects of BMP2 on the growth of liver cancer.

Functions of BMP2 on the survival of liver cancer cells were still controversial. Previous reports indicated that liver cancer cells could respond to BMP2 signaling differently under different conditions (15, 16). M-MDSCs and PMN-MDSCs derived from bone marrows were major myeloid populations associated with tumor development via cytokines IL6, IL8, or IL10 (19, 20). We found that BMP2 could promote liver cancer growth as well as the expansion of MDSCs in mouse models. Thus, BMP2 signaling might promote liver cancer growth indirectly via the activation of MDSCs. Consistently, we found that cytokines secreted by MDSCs could enhance cell proliferation of liver cancer cells H22 and Hepa1-6 (Figures 6A,B). Then, we used the 3D cultures of HCC cells to mimic the tumorigenesis of HCC cells *in vivo*. We found that conditional media from bone marrows induced by BMP2 could enhance the spheres formation of HCC cells Hepa1-6, resulting in more and larger spheres. However, BMPRI inhibitor LDN-193189 could inhibit the effect (Supplementary Figure 4E). The results indicated that BMP2, which induces the expansion of MDSCs in bone marrows, could enhance proliferation and the tumor sphere formation of HCC cells. In addition, we further confirmed that MDSCs could secrete IL6, as shown in Figure 6C. Moreover, intravenous injection of rhBMP2 and implantation of rhBMP2 collagen gels in tumor-burdened mice could increase the IL6 levels in both peripheral blood and tumor tissues (Figures 6D,E and Supplementary Figures 4A,B), indicating that MDSC expansion, induced by BMP2, enhanced liver cancer growth via IL6. We also observed that IL6 could promote cell proliferation of liver cancer cells H22 and Hepa1-6 *in vitro*, and the IL6 neutralized antibody could block the enhancement of cell proliferation and tumor sphere formation of Hepa1-6 induced by MDSC-secreted cytokines (Figures 6F,G and Supplementary Figures 4C–E). Besides, intravenous injection of rhBMP2 and implantation of rhBMP2 collagen gels in tumor-burdened mice could induce higher expression of the cell proliferation marker Ki67 in tumor tissues than the control treatment (Figure 6H). Furthermore, from the public clinical microarray database of TCGA, we found that the expression of BMP2 in liver cancer tissues was associated with Arg1 and IL6, the activation marker of MDSCs (Figures 6I,J). The results demonstrated that BMP2 inducing MDSCs expansion could enhance liver cancer progression via IL6 in human liver cancer as well.

## Discussions

Clinical and non-clinical studies that assessed the association between BMP2 and cancers had come to conflicting conclusions. Several studies reported that BMP2 was a pro-oncogenic protein and could promote the progression of breast cancer, lung cancer, prostate cancer, etc. (31–33), but others also demonstrated that BMP2 inhibited cancer proliferation (34, 35). However, the impact of BMP2 on the malignancy of liver cancers was very rarely investigated. In this study, we found that both intravenous delivery and local implantation of BMP2 could enhance the growth of liver cancers *in vivo*. Moreover, we noticed that increased concentration of BMP2 within the peripheral blood in mice could lead to the expansion of MDSCs in PB and the infiltration of MDSCs into tumor tissues. Consistently with the studies *in vivo*, we found that BMP2 could also lead to the expansion of MDSCs in bone marrow *in vitro*. MDSCs were highly active in the suppression of T-cell response, which subsequently could favor the growth of tumors (36). More importantly, we observed that BMP2 activated MDSCs to secrete IL6, which directly enhanced the proliferation of liver cancer cells. Taken together, these results suggested that high levels of BMP2 could promote the growth progression of liver cancer through the activation of MDSCs (Figure 6K).

Usually, rhBMP2 was delivered locally for promoting bone formation. Thus, it was not likely to have systematic function through the circulation system. However, rhBMP2 was a soluble protein, and an extensive high dose used in clinics to ensure the induction of adequate osteogenesis may lead to blood absorption of rhBMP2. In this study, we found that local implantation of rhBMP2 collagen gels could lead to the increase of blood rhBMP2 concentration in mice, of which the peak value was observed at the second week after implantation, indicating that local delivered rhBMP2 was released into blood. The biological safety of rhBMP2 within the circulation was an important issue that needed to be validated. Lee et al. (37) reported that direct intravenous injection of rhBMP2 at a dosage of 0.5 mg/kg did not lead to adverse effect to healthy rats within 2 weeks of observation. Yoon et al. (38) evaluated the toxicological effect of repetitive intravenous injection of Activin A/BMP-2 in rats, and no observed adverse effects were found. Those studies suggested that rhBMP2 within blood may not have systemic toxicity to normal rats. Nevertheless, in our results, when mice had liver cancers, rhBMP2 administration through both local implantation and intravenous injection could promote the growth of liver cancers. Thus, the safety of rhBMP2 materials used in clinics still needed more concerns.

BMP2 may be a two-edged sword to cancer progression. BMP2 had been reported to have effects on various cancer attributes, including proliferation, invasiveness, metastatic potential, and angiogenesis. BMP2 could induce the proliferation of human breast cancer cells through interacting with type II BMP receptor (39). BMP2 could induce lung cancer migration, invasion, and EMT via activation of MAPK/Runx2/Snail signaling pathway (33). BMP2 had also been reported to play roles in the migration of prostate cancer cells *in vitro* (40). Several other studies came to opposite conclusions that demonstrated that BMP2 could inhibit the proliferation of various cancer cell lines such as MDA-MB-231 breast cancer cells, osteosarcoma cells, and gastric cancer cells (35, 41, 42). However, we found that BMP2 did not promote the proliferation of liver cancer cells directly *in vitro* (Supplementary Figure 5). In summary, the roles that BMP2 plays in tumor progression may be affected by the types of cancer and the microenvironment, which needed future research.

It was important to note that analysis *in vitro* could not represent the full biological effects of BMP2 *in vivo*, as the interaction between BMP2 and cancer environment was more complex *in vivo*. We found that BMP2 within the circulation could promote the expansion of MDSCs in peripheral blood, and the infiltration of MDSCs in tumor, which resulted in the promoted liver cancer growth. MDSCs were one of the major components of the tumor microenvironment. There were two different types of MDSCs: polymorphonuclear MDSC (PMN-MDSC) and monocytic MDSC (M-MDSC). They were generated in the bone marrows and migrated to the tumors to regulate the immune response in cancers (20). They had potent immune suppressive activity that resulted in tumor progression (43). In our study, we treated bone marrow cells from mice with rhBMP2 and found that BMP2 could promote the expansion of both PMN-MDSCs and M-MDSCs *in vitro*. We observed that Smad1/5 in the MDSCs infiltrating tumors was phosphorylated *in vivo*. Moreover, we found that phosphorylated Smad1/5 for bone marrow cells could be induced by BMP2 signaling, while Erk and Stat3 signaling were not activated, indicating that BMP2 signaling might activate MDSCs via canonical Smad1/5/8 signaling rather than non-Smads signaling. MDSCs had been reported to induce T-cell dysfunction in cancer through the production of ROS (44), IL10 (45), and TGF-β (46). There were also other researches reporting that MDSCs enhanced tumor progression via IL6 (47, 48). In our results, BMP2 signaling activated MDSCs to secrete IL6, further enhancing liver cancer cell proliferation.

Our report was the first to provide data indicating that rhBMP2 could promote the progression of liver cancers through activating MDSCs indirectly. However, we only analyzed two hepatocellular carcinoma cell lines; other liver cancer subtypes, like cholangiocarcinoma or the mixed type, were not analyzed, which needed further research. Moreover, we only studied the dosage and carriers of rhBMP2 that could lead BMP2 to be released into the blood; whether locally delivered rhBMP2 without blood release could promote the growth of ectopic tumor could not be speculated. Further research using more rhBMP2 delivery modes and different dosages is therefore necessary.

## Conclusions

In conclusion, increased concentration of BMP2 within the peripheral blood could promote the growth of liver cancers *in vivo*. In addition, BMP2 could accelerate cancer growth through inducing the expansion of MDSCs in bone marrows. Those effects may be mediated by the response of bone marrow cells to canonical BMP2 signaling pathway. BMP2 activated MDSCs to secrete IL6, further enhancing liver cancer cell proliferation. Our findings demonstrated that high levels of BMP2 could enhance liver cancer growth via regulating immune cells in the tumor microenvironment, like MDSCs.

## Data Availability Statement

Publicly available datasets were analyzed in this study. This data can be found here: the TCGA Research Network: https://www.cancer.gov/tcga and Oncomine Research Network: https://www.oncomine.org.

## Ethics Statement

The animal study was reviewed and approved by Animal Ethical Committee of Fujian Medical University (2018-039). Written informed consent was obtained from the individual(s) for the publication of any potentially identifiable images or data included in this article.

## Author Contributions

GW: conceptualization, data curation, formal analysis, funding acquisition, and writing original draft. FH: conceptualization, data curation, formal analysis, writing, review, and editing. YC: data curation. YZ: investigation. YH: investigation. YX: supervision and investigation.

### Conflict of Interest

The authors declare that the research was conducted in the absence of any commercial or financial relationships that could be construed as a potential conflict of interest.
