# Supplementary material for: High Levels of BMP2 Promote Liver Cancer Growth via the Activation of Myeloid-Derived Suppressor Cells
Source: Front Oncol. 2020 Mar 4;10:194. doi: 10.3389/fonc.2020.00194 (PMC7064622; doi:10.3389/fonc.2020.00194)

Supplementary Material

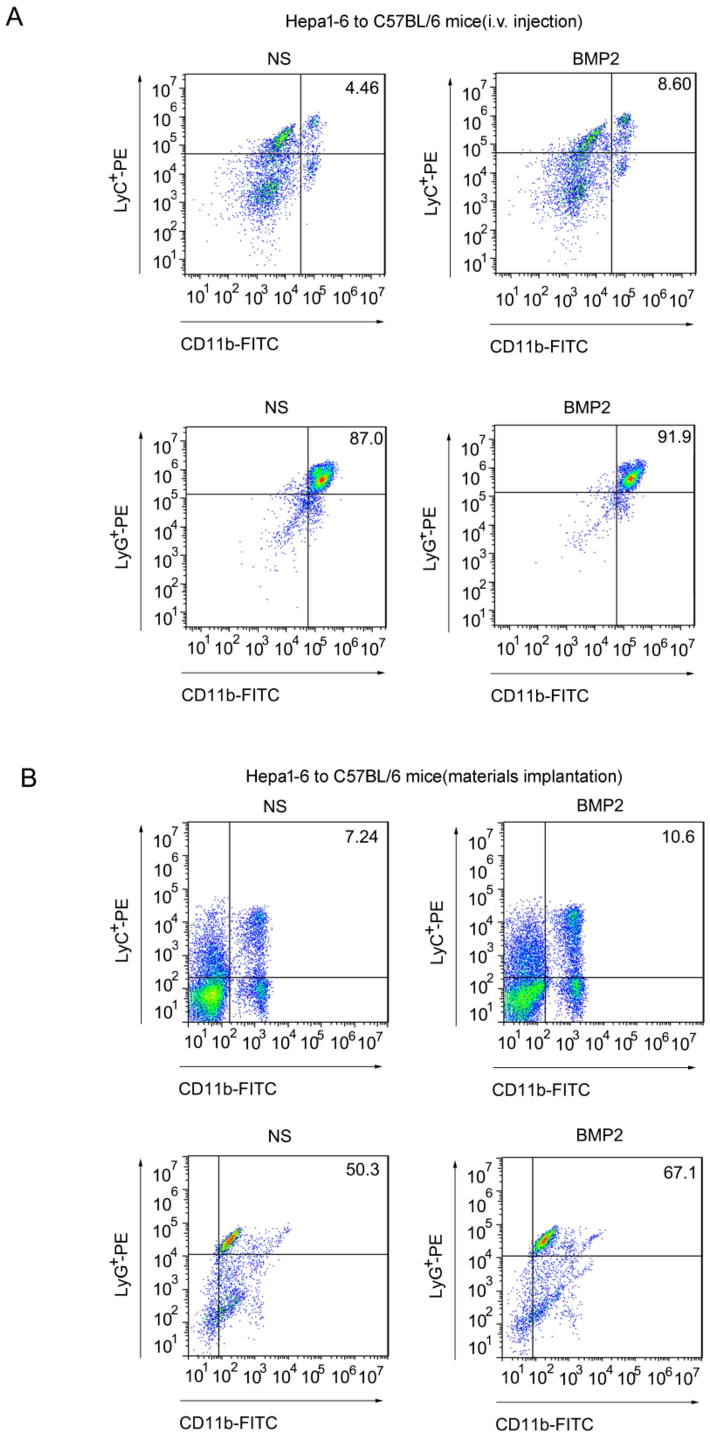

Supplementary Figure 1. BMP2 promoted the expansion of MDSCs in peripheral blood.

(A) Represent flow cytometric images of monocyte derived MDSCs and polymorphonuclear derived MDSCs from rhBMP2 solution intravenously injected C57BL/6 mice.

(B) Represent flow cytometric images of monocyte derived MDSCs and polymorphonuclear derived MDSCs from rhBMP2 collagen gels implanted injected C57BL/6 mice.

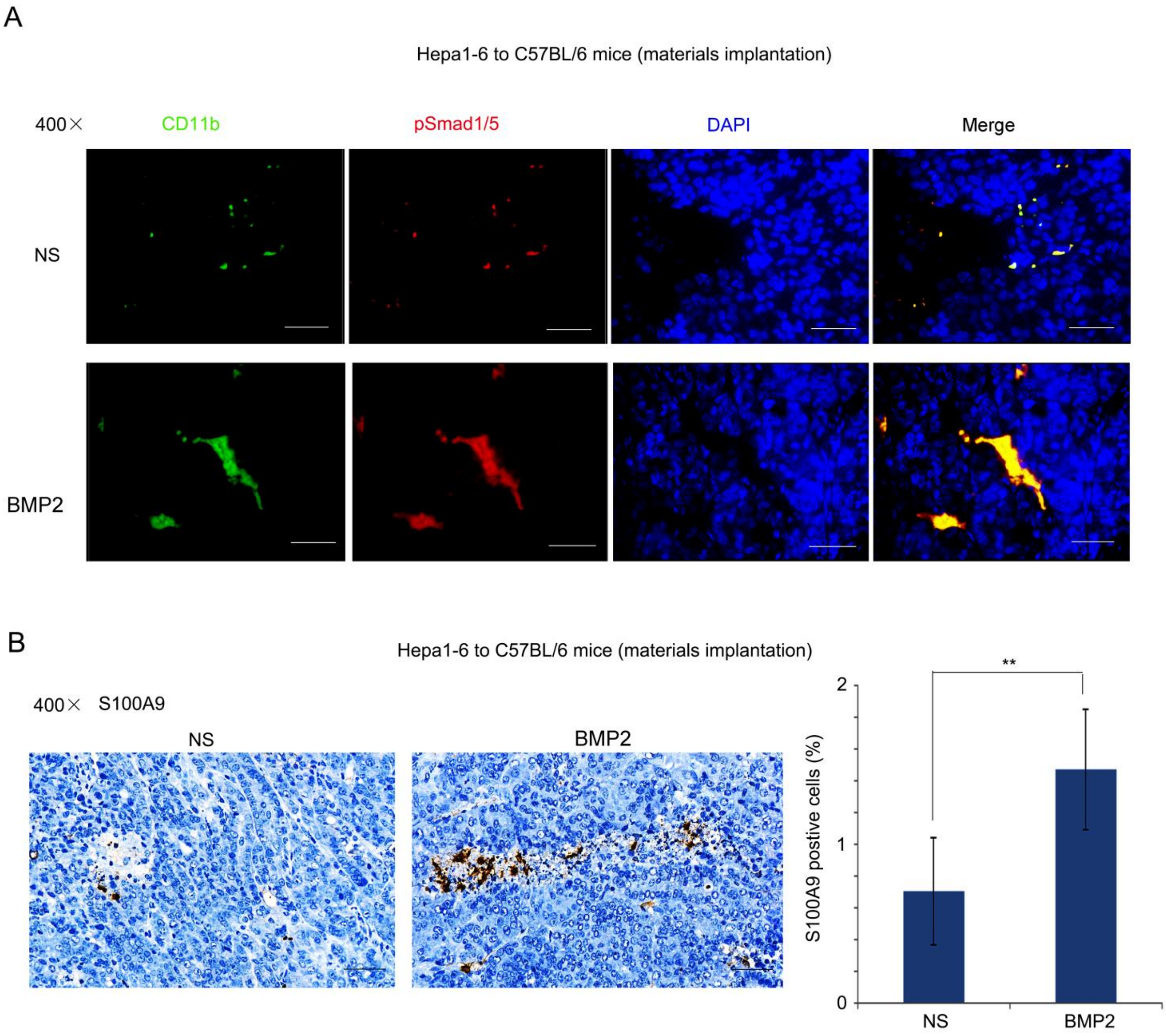

Supplementary Figure 2. BMP2 promoted the infiltration of MDSCs into Hepa1-6 tumors.

(A) Immunofluorescence images of subcutaneous Hepa1-6 tumor samples from rhBMP2 collagen gels implanted ICR mice. Blue: DAPI, Green: CD11b-FITC, Red: Smad1/5/8-TRITC. Scale bar: 100μm.

(B) Immunohistochemistry images indicated the expression of S100A9 of subcutaneous Hepa1-6 tumor samples from rhBMP2 collagen gels implanted ICR mice. Scale bar: 50μm. Average percentages of S100A9 positive cells of at least three fields were shown on the right. **: $P<0.01$.

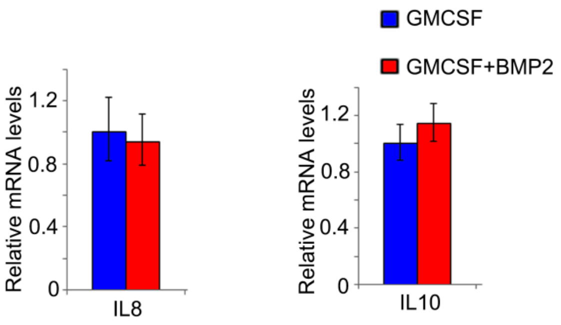

Supplementary Figure 3. MDSCs activated by BMP2 did not secret IL8 or IL10.

Comparison of relative IL8 and IL10 mRNA levels of bone marrow cells treated with BMP2. *: *P<0.05*, **: *P<0.01*

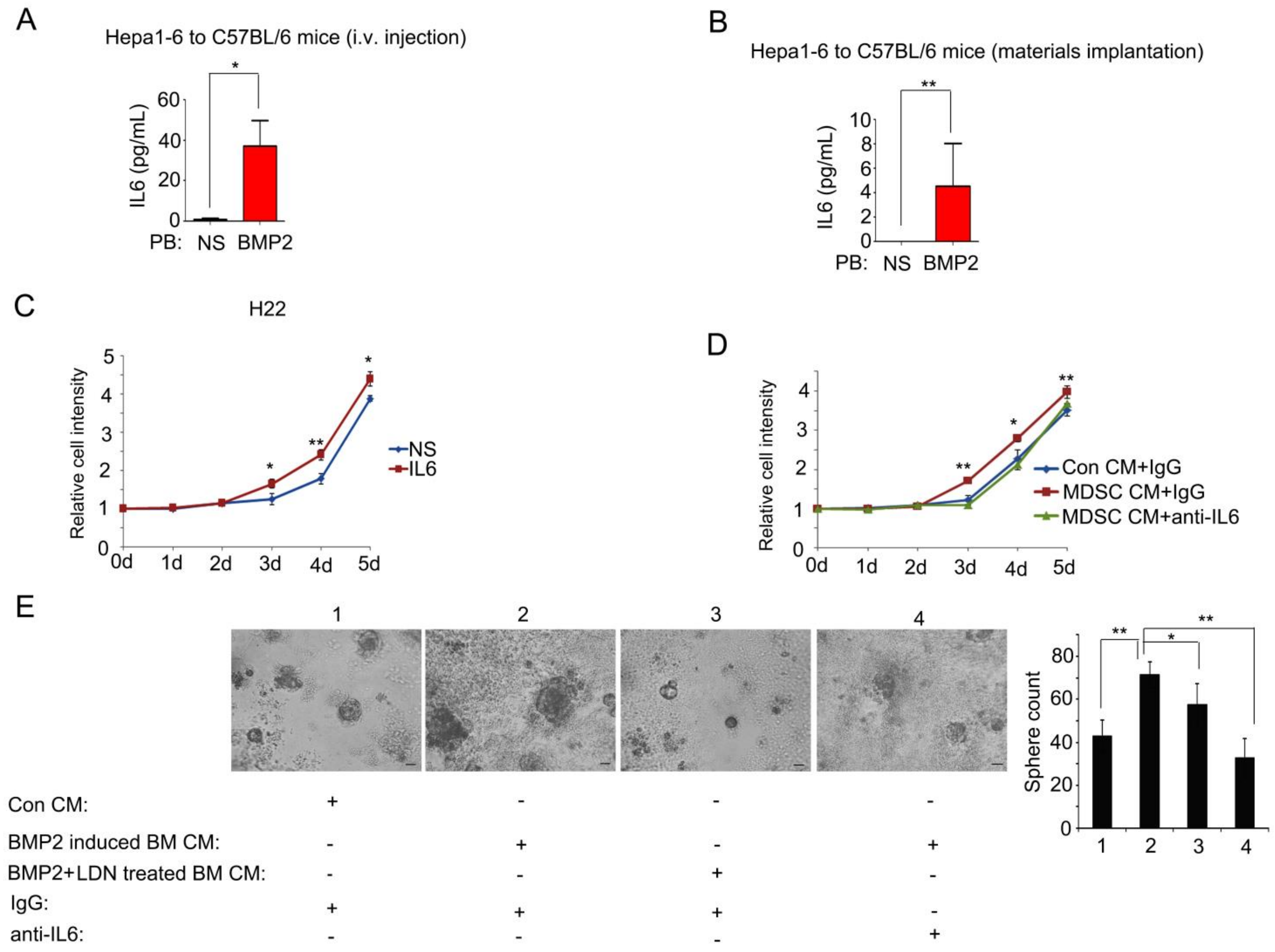

Supplementary Figure 4. BMP2 inducing MDSCs expansion enhanced liver cancer growth via IL6.

(A) IL6 concentration in peripheral blood of rhBMP2 solution intravenously injected C57BL/6 mice for NS and rhBMP2 groups were compared. *: *P<0.05*, **: *P<0.01*

(B) IL6 concentration in peripheral blood of rhBMP2 collagen gels implanted C57BL/6 mice for NS and rhBMP2 groups were compared. *: *P<0.05*, **: *P<0.01*

(C) About 3,000 H22 cells were cultured in each well of 96 well plates with vehicle or IL6. Relative cell intensity was measured with a cell-counting kit after indicated time. *: *P<0.05*, **: *P<0.01*

(D) About 3,000 H22 cells were cultured in each well of 96 well plates with indicated treatment. Relative cell intensity was measured with a cell-counting kit after indicated time. *: $P<0.05$, **: $P<0.01$

(E) Hepa1-6 cells of different treatment were cultured in matrigel to form spheres. Photos were taken after six days. Scale bars, 10μM.

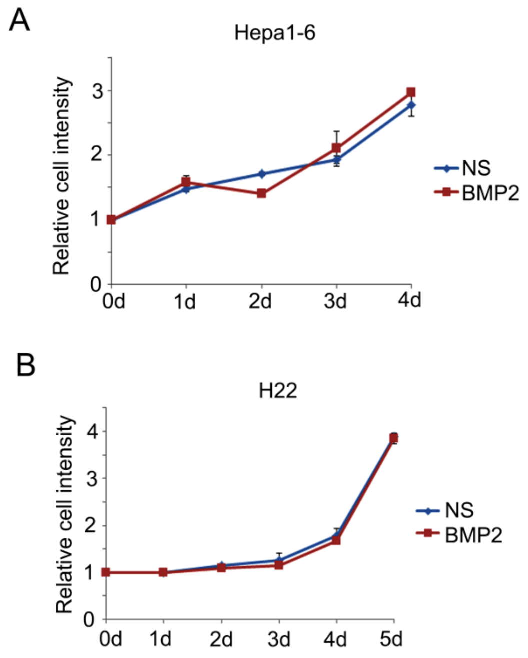

Supplementary Figure 5. BMP2 did not affect the proliferation of HCC cells directly.

(A) About 3,000 Hepa1-6 cells were cultured in each well of 96 well plates with vehicle or BMP2. Relative cell intensity was measured with a cell-counting kit after indicated time. *: *P<0.05*, **: *P<0.01*

(B) About 3,000 H22 cells were cultured in each well of 96 well plates with vehicle or BMP2. Relative cell intensity was measured with a cell-counting kit after indicated time. *: *P<0.05*, **: *P<0.01*

Original gels for Fig 5C

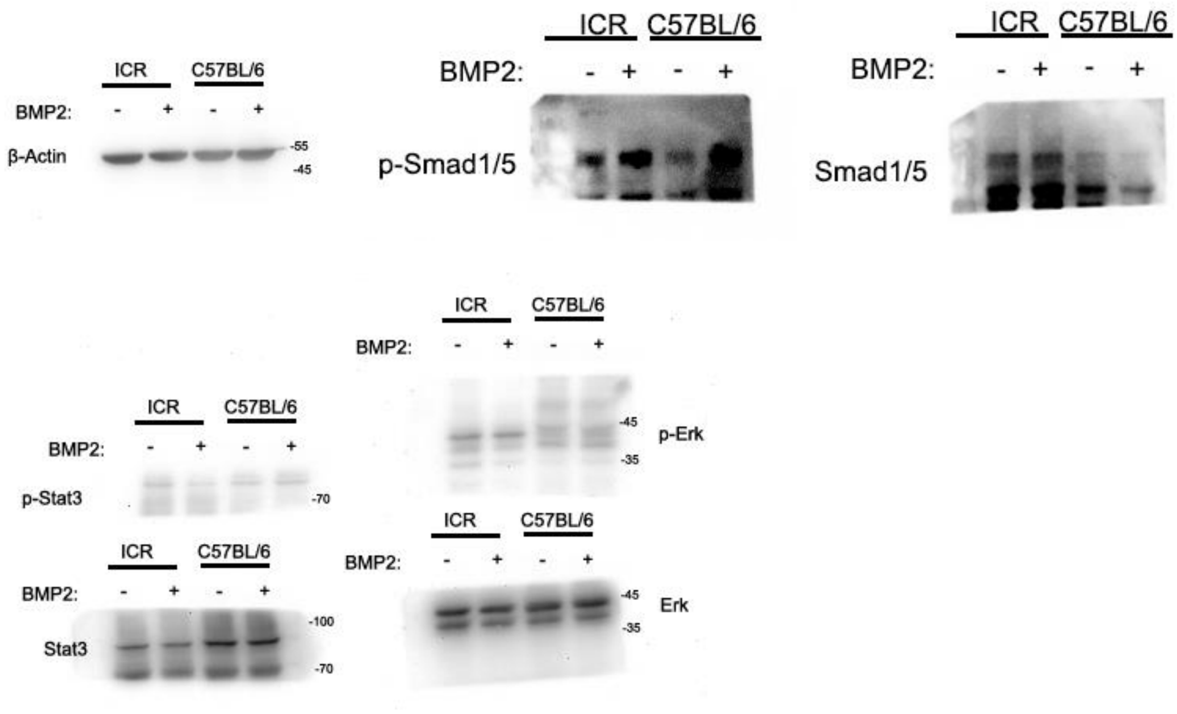

Supplement: Supplementary file 1 [file Data_Sheet_1.pdf]
